# Investigating the Effects of Demographic Factors on the Prevalence of Cutaneous Leishmaniasis in a Focus Area of Northeastern Iran

**DOI:** 10.1155/jotm/7537793

**Published:** 2025-09-04

**Authors:** Fateme Rajabi Gharaii, Mitra Boroomand, Amin Mohammadpour, Mohadeseh Motamed-Jahromi, Aboozar Soltani

**Affiliations:** ^1^Department of Environmental Health Engineering, School of Health, Student Research Committee, Shiraz University of Medical Sciences, Shiraz, Iran; ^2^Department of Biology and Control of Vector Borne Diseases, Health School, Shiraz University of Medical Science, Shiraz, Iran; ^3^Research Center for Social Determinants of Health, Jahrom University of Medical Sciences, Jahrom, Iran; ^4^Department of Public Health, School of Health, Fasa University of Medical Sciences, Fasa, Iran; ^5^Research Center for Health Sciences, Institute of Health, Department of Medical Entomology and Vector Control, School of Health, Shiraz University of Medical Sciences, Shiraz, Iran

**Keywords:** clinical features, cutaneous leishmaniasis, demographic factors, incidence, Iran, prevalence, public health

## Abstract

Iran is a significant center for cutaneous leishmaniasis, making it crucial to identify transmission modes and control measures to improve public health. Due to the frequent cases of leishmaniasis at health centers in Neyshabur and Firuzeh, this study aimed to create a comprehensive profile of the demographic and environmental factors influencing the prevalence of this condition. This retrospective study utilized secondary data involving 807 patients with suspected cutaneous leishmaniasis, referred to various health facilities, including clinics, outpatient centers, and hospitals in Neyshabur and Firuzeh Counties between 2013 and 2019. Of the total patients, 47.6% were male and 52.4% were female. The highest disease incidence was observed in middle-aged individuals, with 68% of cases occurring in urban areas and the remainder in rural settings. Of those infected, roughly 55% had no recent travel history, while approximately 45% reported recent travel. Among the subjects studied, 58.73% had one wound, 17.22% had two, and 7.08% had five or more. The hand was the most affected area, representing 20.69% of cases, followed by the face at 19.21%. A statistical correlation was found between patients' place of residence and occupational group with the type of cutaneous leishmaniasis. The incidence of the disease declined in 2018, but Neyshabur's central districts showed a higher average annual incidence rate than other studied areas, indicating increased risk for residents contracting cutaneous leishmaniasis. This highlights the need for targeted education for at-risk populations to reduce incidence rates and prevent disease spread.

## 1. Introduction

Health is a fundamental human right, and healthcare is crucial for improving the quality of life [1, 2]. Despite progress in controlling infectious diseases, parasitic diseases remain a major health problem [3]. Around 75% of emerging infectious diseases originate from animals, making them zoonotic. Their emergence results from a complex mix of human activities, genetic changes, environmental shifts, social conditions, and climate-related factors [4]. These diseases have severely impacted healthcare systems, imposed significant economic burdens worldwide, and led to the loss of countless lives [5].

Leishmaniasis is a vector-borne skin disease caused by different intracellular protozoa of the genus *Leishmania* and is found in certain geographical areas [6]. This zoonotic disease affects both humans and animals, presenting in three forms in Iran: rural cutaneous leishmaniasis (CL), urban CL, and visceral leishmaniasis, caused by *Leishmania major*, *Leishmania tropica*, and *Leishmania infantum*, respectively [7]. CL is found worldwide, particularly in underdeveloped and developing countries. Due to its health significance, the World Health Organization (WHO) has introduced it as one of six major diseases in tropical and subtropical regions [8]. A comprehensive WHO report highlights the widespread nature of leishmaniasis across 98 countries, indicating that over 350 million people are at significant risk of this infectious disease [9]. The burden of CL is disproportionately concentrated in six countries: Afghanistan, Algeria, Brazil, Colombia, Iran, and Syria, which account for approximately 70%–75% of global cases [10]. The primary foci of CL are mainly located between 28 and 42° north latitude [11].

The incubation period for the disease typically ranges from 2 to 8 weeks, though it can be longer [12]. A previous study highlighted the metropolitan areas of Shiraz, Mashhad, and Isfahan, along with the provinces of Golestan, Kerman, Khuzestan, Ilam, Yazd, Sistan and Baluchistan, Semnan, Qom, North Khorasan, and Bushehr, as having the highest infection rates [13]. The notable increase in CL infection centers and rates can be attributed to a combination of environmental, socioeconomic, behavioral, and demographic factors [14]. The spread of leishmaniasis is closely linked to anthropogenic activities such as agricultural developments, migration of nonimmune individuals into endemic areas, and rapid urbanization without proper planning [15]. In addition, geography and climate favor the proliferation of rodents and sandflies that transmit the disease [16].

Symptoms of CL include ulcers on the body, face, hands, and feet, which can persist for up to a year. While these ulcers typically heal spontaneously, they result in permanent scarring [17]. Given the frequent cases reported at Neyshabur health centers and the lack of recent comprehensive studies, there is an urgent need for epidemiological research to clarify the current situation. This study aimed to address this gap by systematically investigating the distribution and determinants of CL in Neyshabur and Firuzeh Counties over a six-year period from 2013 to 2019.

## 2. Methods

**Figure 1:**
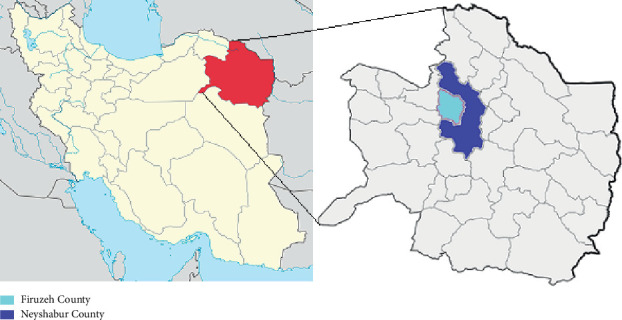
Iran's map indicating the study areas (Neyshabur and Firuzeh Counties, Razavi Khorasan Province).

Located in northeastern Iran, Neyshabur and Firuzeh have cold winters and hot summers, with a semiarid climate characterized by low precipitation and high evaporation rates. Agriculture is the primary livelihood in these counties, with wheat, barley, and cotton as the main crops, while handicrafts, particularly carpet weaving, also play a significant role in the local economy (Figure 1). This retrospective study involved 807 individuals suspected of CL, who sought medical attention at health facilities, including clinics, outpatient centers, and hospitals in Neyshabur and Firuzeh Counties. The study period spanned six years, from April 2013 to December 2019.

In this study, data were meticulously recorded on patient forms, including the year and month of report submission, residential area, housing type, patient age, history of scars, recent familial illness, lesion location and size, lesion count, treatment regimen, and patient age group. Demographic analysis of the patient data was conducted using the Statistical Package for the Social Sciences (SPSS) (Version 23). All information was obtained from the records of Neyshabur University of Medical Sciences. ArcGIS (v: 10.8) was used for the spatial distribution of CL cases.

## 3. Results

**Figure 2:**
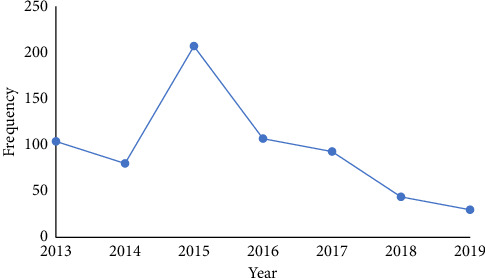
Annual trends in the prevalence of cutaneous leishmaniasis in Neyshabur and Firuzeh Counties, Razavi Khorasan Province, Iran.

**Figure 3:**
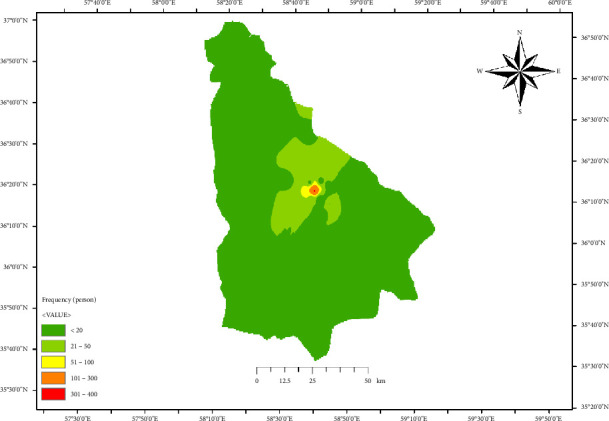
Geospatial distribution of cutaneous leishmaniasis cases in the studied areas of northeastern Iran.

**Figure 4:**
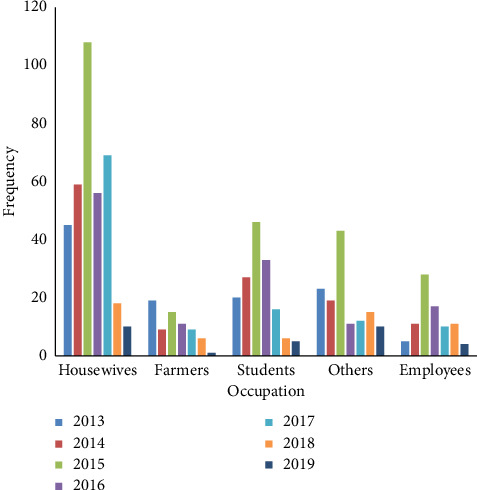
Occupational stratification of leishmaniasis patients.

The annual trend of CL prevalence in Neyshabur and Firuzeh Counties, located in Razavi Khorasan Province, Iran, is illustrated in Figure 2.  Figure 3 shows the demographic distribution of CL cases in Neyshabur and Firuzeh Counties. The highest prevalence of CL cases was observed among housewives, comprising 45.2% of cases, while farmers had the lowest prevalence at 8.7% (Figure 4).

Table 1 indicates that 68% of patients reside in urban areas, while 32% are from rural regions. Females account for 52.4% of cases, and males for 47.6%. The highest incidence is among middle-aged individuals, totaling 332 (41%), followed by adolescents at 193 (24%). Approximately 55% of infected individuals had no travel history, while about 45% reported traveling. The disease incidence increases as temperatures drop, starting in early autumn and peaking in January. Microscopic examination indicated that most smears were positive for *Leishmania tropica*.

Among the subjects studied, 58.73% had a single wound, 17.22% had two wounds, and 7.08% had five or more. The hand was the most affected area, accounting for 20.69% of cases, followed by the face at 19.21%. Other body parts were also affected (see Table 2 for details).

## 4. Discussion

This study aimed to analyze the epidemiological patterns of leishmaniasis in Neyshabur and Firuzeh Counties, particularly within the jurisdiction of Neyshabur University of Medical Sciences. In Iran, the incidence of CL has been rising, with new endemic regions recently identified [16]. This research seeks to illuminate the current situation in these areas and enhance the understanding of CL epidemiology. Notably, the incidence of the disease showed a decline in 2018 and 2019 during the seven-year study period. Sandfly–borne infections are shaped by factors such as climate change, poor living conditions, migration, and conflict [18]. Staying indoors during periods of peak sandfly activity reduces exposure to these vectors and consequently decreases the risk of bites. Hussain et al. found a strong link between CL and various demographic, occupational, and socioeconomic factors. The most common risk factor, reported in 66% of cases, was the lack of protective measures such as insect repellents, bed nets, and protective clothing, highlighting their critical role in preventing disease transmission [19].

The results show that the highest infection rates occur in the middle-aged group, with women experiencing higher rates than men. This suggests that middle-aged individuals, particularly women, may lack awareness of protective measures against sand fly vectors. A related study in Marvdasht found that those aged 15–30 were the most affected, highlighting the importance of age demographics in disease prevalence [20]. In Isfahan Province, cases were increased among individuals aged 10–20, while Kashan reported the majority of cases among the 20–29 age group [21, 22]. These findings underscore the varying age distributions across regions and the necessity of considering local demographics and epidemiological factors in disease research and management. In the study by Hatami et al. in Aran and Bidgol, 60.6% of patients were male, with the remainder female. The most common occupations were housewives, students, and workers. A majority (88.8%) of cases were from rural areas. Only 0.8% had a personal history of previous infection, and 43.6% had a history of travel [23].

This investigation found a higher prevalence of CL among women, accounting for 52% compared to 48% in men. This aligns with several studies from different regions, such as Amraee's research in Poldakhter, which reported a prevalence of 54.19% among women, and Khosrotaj's study in Dezful, which found a rate of 54.7% [24, 25]. These findings collectively confirm that women bear a greater burden of CL infection than men in these areas. One possible explanation for this discrepancy is that women engage in economic activities such as carpet weaving, often in poorly lit rooms or basements that are conducive to vector habitats. CL is influenced by cultural and occupational factors, leading to varied outcomes. Men, who typically work outdoors, may have received more information about the disease from health professionals, resulting in lower exposure to sand fly bites. In addition, men may seek treatment less frequently, leading to fewer recorded cases. It is important to note that this study's findings contrast with those of Sanei-Dehkordi and colleagues in Hormozgan, Jorjani in Golestan, and Akhlagh in Hamadan, highlighting the need for further research into the complexities of CL epidemiology and its connection to gender and occupational factors [26–28]. The study by Abbaszadeh reported that leishmaniasis was most prevalent in rural areas of Sabzevar County (78.9%), followed by urban areas (21%), with the lowest rates observed in nomadic populations. Overall, 63% of cases were male and 37% were female [29]. The findings highlight the need for health policymakers to prioritize education and awareness among domestic caregivers and students. Given the incidence of CL in Neyshabur and the absence of travel to disease-prone areas among those affected, it is evident that this city is endemic, providing suitable conditions for sandfly breeding. This suggests an active disease cycle in the region. In addition, factors such as housing characteristics and urban or rural residency influence disease contraction. The study found a higher number of patients from urban areas compared to rural ones. Some rural residents near the city keep domestic animals, creating environments conducive to sandfly reproduction.

In Neyshabur, housewives represent 45.2% of those affected by CL. This is likely due to their prolonged time at home and insufficient attention to protective measures, such as installing nets on doors and windows, which increases their vulnerability to CL infection. Sandflies are known to bite women and children more frequently. In addition, women may be more motivated to seek diagnosis and treatment at health centers due to concerns about appearance, their children's health, and the impact of wounds. A study in southwestern Iran found that students had the highest incidence of CL [30]. Neyshabur has a dry and hot climate, with CL typically emerging in early autumn and peaking in January. This delay is linked to daily temperatures and the cumulative temperature effect that correspond with the disease's incubation period. Symptoms usually appear at least 2 months after a sandfly bite. The highest temperatures in Neyshabur occur in July and August, the prime period for vector activity. Therefore, the onset of the disease in autumn is likely due to bites from the preceding 2 months. The decline in cases during spring is attributed to decreased sand fly activity in winter, which is marked by unfavorable climatic conditions for vectors. Statistical analysis shows that most cases in January and February occur in winter, while the fewest cases are reported in July during summer. These patterns align with findings from a study by Rassa and colleagues in Dasht-e Azadegan County [31]. The findings of this study indicate that the hand is the most common site for CL lesions. Over half of the patients had a single ulcer, while 17.22% had two ulcers and 7.08% had five. Rassa's study supports these results, with over half of the subjects showing a single ulcer [31]. Since the leishmaniasis vector cannot bite through clothing, it primarily affects exposed areas, such as the hands, feet, and face [26]. In Shaikh's study conducted in Karachi, 96.3% of CL lesions were located on exposed parts of the body. Most patients (64.2%) had a single lesion, 19.6% had two, and one patient presented with seven lesions [32]. A study on CL in Gonabad, Bardaskan, and Kashmar (Central Khorasan) found that lesions were most commonly located on the hands (62.75%), followed by the head and neck (24.8%) and the body (2.7%) [33]. The study by Hamzavi on CL in Qasr-e Shirin County showed that most patients (45.6%) had a single lesion or scar, while 17.4% had five or more [34].

## 5. Conclusion

To effectively control CL, a combination of strategies is recommended. Since many climatic factors are difficult to change, educating the specific demographic groups most affected by the disease is crucial. Key measures include controlling rodent reservoirs, assessing key parameters influencing disease incidence, and using both standard and insecticide-treated nets to limit its spread. In addition to health education, essential prevention efforts involve improving both urban and rural environments, regulating dogs and stray animals, and ensuring health officials actively monitor and manage the disease vectors and reservoirs in the area.

## Figures and Tables

**Table 1:** Number of cutaneous leishmaniasis cases by demographic information in the studied areas of northeastern Iran.

Demographic factors		Frequency	Percent (%)
Sex	Female	423	52.4
	Male	384	47.6

Age	Child	63	8
	Adolescent	193	24
	Young	121	15
	Middle-aged	332	41
	Old	98	12

Residential location	City	547	68
	Rural	260	32

Travel history	Yes	361	45
	No	446	55

**Table 2:** Distribution of the frequency of cases of cutaneous leishmaniasis according to the location and number of lesions.

Variables	Location and number of lesions	Frequency (%)
Head and neck	Face	19.21
	Neck	3.35

Hand	Hand	20.69
	Forearm	15.12
	Arm	5.95

Trunk	Trunk	1.36

Leg	Leg	4.36
	Feet	5.82

Other things	Combinations	< 3

Wounds	One wound	58.72
	Two wounds	17.22
	Three wounds	6.94
	Four wounds	3.09
	Five wounds	7.08

## Data Availability

The data that support the findings of this study are available on request from the corresponding author.
